# Perceived Algorithmic Control and Work Engagement in Platform Gig Work: Asymmetric Pathways via Role Stress and Emotional Exhaustion

**DOI:** 10.3390/bs16081392

**Published:** 2026-08-13

**Authors:** Chunna Shi, Di Zhang, Junping Ma, Jianping Hou, Li Cao, Yong Yang

**Affiliations:** School of Economics and Management, Xi’an Technological University, Xi’an 710021, China

**Keywords:** platform gig work, perceived algorithmic control, role stress, emotional exhaustion, work engagement, overall social support, food-delivery workers

## Abstract

Algorithmic management is central to platform gig work, but its distinct forms of control may relate differently to worker strain. We examine these relationships in food delivery, a typical form of platform gig work. In this setting, algorithms continuously allocate tasks, track performance, evaluate workers, and shape behavior. The Stressor–Strain–Outcome (S-S-O) model provides the overarching framework. The Job Demands–Resources (JD-R) perspective explains why the three control dimensions may show different associations, while cognitive appraisal theory provides a supplementary lens. Specifically, the study links standardized guidance, tracking evaluation, and behavioral constraints to work engagement through role stress and emotional exhaustion, with overall social support as a moderator. Survey data from 608 food-delivery workers were analyzed using partial least squares structural equation modeling (PLS-SEM) with 5000 bootstrap resamples. Standardized guidance was negatively associated with role stress and emotional exhaustion, whereas tracking evaluation and behavioral constraints were positively associated with both. Role stress and emotional exhaustion each carried significant indirect associations with work engagement; the serial indirect associations were significant but small. Overall social support strengthened the positive associations of tracking evaluation and behavioral constraints with both strain variables. For standardized guidance, however, higher support strengthened, rather than weakened, the negative associations with strain. Predictive assessment, control-variable models, and covariance-based SEM sensitivity analyses produced broadly consistent findings. These results show that distinct forms of algorithmic control relate differently to strain. Because the data are cross-sectional and were collected from food-delivery workers, causal and temporal inferences, as well as generalization across occupations, remain limited.

## 1. Introduction

Digital labor platforms use algorithms to allocate tasks, monitor performance, evaluate workers, and administer rewards and penalties. We examine platform gig work in the food-delivery context. Food-delivery workers interact with platform systems on every order: algorithms allocate orders, provide navigation, set time expectations, track performance, and administer rewards or penalties. Because this exposure is both continuous and intensive, food delivery offers a useful setting for studying perceived algorithmic control. It should not, however, be regarded as representative of all platform occupations (Chen, 2020; Duggan et al., 2020; Pei et al., 2021; Wood et al., 2019).

Algorithmic management was already a well-established line of inquiry before 2024, and the literature presents a mixed picture. Earlier studies examined how platform algorithms shape autonomy, task allocation, and perceived control (Duggan et al., 2020; Pei et al., 2021; Wood et al., 2019). More recent studies, including work published since 2024, associate intensive monitoring and control with psychosocial risk, resource depletion, turnover intention, and lower well-being (B. Liang et al., 2025; Mbare et al., 2024; Y. Wang et al., 2024; Yu et al., 2025; Zhang et al., 2026). At the same time, clear guidance, useful feedback, and role clarity may provide valuable work resources. Research on work engagement likewise points to both a job-clarity pathway and an autonomy-restriction pathway (Deng et al., 2024). Together, these findings suggest that outcomes depend less on the presence of algorithms than on the functions they perform.

Pei et al. (2021) conceptualized perceived algorithmic control in terms of standardized guidance, tracking evaluation, and behavioral constraints. Subsequent studies linked these dimensions to emotional exhaustion, overwork, turnover intention, and work engagement. Standardized guidance often showed a different pattern from tracking evaluation and behavioral constraints (Pei et al., 2024; Sun et al., 2024; Yu et al., 2025; Zhang et al., 2024). Qualitative research also shows that food-delivery workers use different coping strategies when they face algorithmic management threats (Weber et al., 2026). Yet few studies compare all three dimensions within a single model that jointly considers role stress, emotional exhaustion, work engagement, and overall social support.

This study uses the S-S-O model (Koeske & Koeske, 1993) as its organizing framework. We treat the three dimensions of perceived algorithmic control as focal work conditions. Role stress and emotional exhaustion are distinct strain responses, and work engagement is the outcome. The JD-R perspective (Demerouti et al., 2001) provides the main explanation for why standardized guidance may function as a resource, whereas tracking evaluation and behavioral constraints may function as demands. Cognitive appraisal theory (Lazarus & Folkman, 1984) is used only as a supplementary lens to help explain why workers may interpret the same algorithmic feature differently. Because appraisal stages were not measured, role stress and emotional exhaustion are not treated as proxies for primary and secondary appraisal.

This study makes four specific contributions. First, it compares the directions of the associations across three established dimensions of algorithmic control within a single model. Second, it considers role stress and emotional exhaustion together in relation to work engagement. Third, it examines overall social support as a boundary condition that may not always act as a buffer. Fourth, it provides evidence on platform gig work from an algorithmically intensive sample of food-delivery workers in China. The study does not claim to introduce the three-dimensional conceptualization, establish a psychological process that unfolds over time, or show that the findings generalize to all platform occupations.

## 2. Literature Review and Hypotheses

### 2.1. Integrated Theoretical Framework

The S-S-O model (Koeske & Koeske, 1993) provides the overall structure for the study. The stressor component comprises the three dimensions of perceived algorithmic control as externally imposed features of platform work. Role stress and emotional exhaustion are treated as distinct strain responses, and work engagement is the focal outcome. Referring to these work conditions as stressors does not mean that every algorithmic feature is harmful. Rather, the model identifies the work conditions whose associations with strain are examined.

The JD-R perspective (Demerouti et al., 2001) explains why these associations may differ. Job demands require sustained effort and carry psychological costs, whereas job resources help people complete tasks, manage demands, or achieve work goals. Standardized guidance may provide clarity, navigation, and task coordination. It is therefore treated as resource-like. Tracking evaluation and behavioral constraints increase surveillance, time pressure, accountability, and restrictions on discretion. They are therefore treated as demand-like. This distinction underpins H1–H6.

Cognitive appraisal theory (Lazarus & Folkman, 1984) provides a secondary lens. It suggests that workers’ responses depend partly on how they interpret algorithmic requirements and the coping resources available to them. Appraisal itself was not measured. Role stress is not equated with primary appraisal, nor is emotional exhaustion equated with secondary appraisal. Moreover, the cross-sectional data cannot establish a two-stage process over time. The proposed ordering should therefore be understood as theory-guided rather than temporal.

### 2.2. Perceived Algorithmic Control Dimensions and Strain

Perceived algorithmic control refers to workers’ awareness of how platform algorithms organize and regulate labor through standardized guidance, tracking evaluation, and behavioral constraints (Pei et al., 2021). Role stress includes ambiguity, conflict, and overload that arise when work expectations are unclear, incompatible, or beyond a worker’s capacity (Cui et al., 2014; Gilboa et al., 2008; Rizzo et al., 1970). Emotional exhaustion refers to the loss of emotional and energetic resources under sustained work demands (Maslach et al., 2001). We expect the three control dimensions to exhibit different associations with these two forms of strain.

Standardized guidance provides instructions, route information, and cues about task allocation. It may lessen role ambiguity by clarifying what workers should do and reducing the effort required for planning and coordination. In turn, this clarity may conserve cognitive and emotional resources (Chen, 2020; Pei et al., 2021; Pei et al., 2024). In JD-R terms, standardized guidance may therefore function primarily as a job resource.

**Hypothesis 1 (H1).**  *Perceived algorithmic standardized guidance is negatively associated with food-delivery workers’ role stress.*

**Hypothesis 2 (H2).**  *Perceived algorithmic standardized guidance is negatively associated with food-delivery workers’ emotional exhaustion.*

Tracking evaluation involves continuous monitoring of location, progress, speed, and performance. This persistent visibility may increase accountability, reduce discretion, and sustain time pressure. These pressures may be especially pronounced when algorithmic assessments conflict with workers’ situational judgments (Mbare et al., 2024; Pei et al., 2024; Sun et al., 2024). Tracking evaluation is therefore treated as a job demand associated with greater role-related and emotional strain.

**Hypothesis 3 (H3).**  *Perceived algorithmic tracking evaluation is positively associated with food-delivery workers’ role stress.*

**Hypothesis 4 (H4).**  *Perceived algorithmic tracking evaluation is positively associated with food-delivery workers’ emotional exhaustion.*

Behavioral constraints rely on rankings, rewards, penalties, and compulsory performance rules to encourage self-regulation. Food-delivery workers may simultaneously face platform and customer expectations, income consequences, and pressure to complete orders with limited time or rest (Pei et al., 2024; Sun et al., 2024; Zhan et al., 2023). Together, these conditions may create role conflict and overload and consume emotional resources.

**Hypothesis 5 (H5).**  *Perceived algorithmic behavioral constraints are positively associated with food-delivery workers’ role stress.*

**Hypothesis 6 (H6).**  *Perceived algorithmic behavioral constraints are positively associated with food-delivery workers’ emotional exhaustion.*

### 2.3. Independent Indirect Associations Through Role Stress and Emotional Exhaustion

Role stress and emotional exhaustion are related but distinct pathways of strain. Role stress reflects difficulty in understanding, reconciling, or meeting role expectations, which may divert attention and motivation from active involvement in work (X. Li & Xia, 2022; C. Wang & Zhu, 2018). Emotional exhaustion reflects depleted affective and energetic capacity. Such depletion is difficult to reconcile with the vigor and dedication required for work engagement (Maslach et al., 2001; Yao & Zhang, 2020; Zhang et al., 2024). Each dimension of perceived algorithmic control may therefore be indirectly associated with work engagement through either form of strain.

**Hypothesis 7a (H7a).**  *Role stress carries an indirect association between perceived algorithmic standardized guidance and food-delivery workers’ work engagement.*

**Hypothesis 7b (H7b).**  *Role stress carries an indirect association between perceived algorithmic tracking evaluation and food-delivery workers’ work engagement.*

**Hypothesis 7c (H7c).**  *Role stress carries an indirect association between perceived algorithmic behavioral constraints and food-delivery workers’ work engagement.*

**Hypothesis 8a (H8a).**  *Emotional exhaustion carries an indirect association between perceived algorithmic standardized guidance and food-delivery workers’ work engagement.*

**Hypothesis 8b (H8b).**  *Emotional exhaustion carries an indirect association between perceived algorithmic tracking evaluation and food-delivery workers’ work engagement.*

**Hypothesis 8c (H8c).**  *Emotional exhaustion carries an indirect association between perceived algorithmic behavioral constraints and food-delivery workers’ work engagement.*

### 2.4. Serial Indirect Association

The S-S-O framework also allows for a serial association in which role-related strain is linked to emotional depletion, which is in turn associated with work engagement. Repeatedly dealing with unclear, conflicting, or excessive expectations may drain emotional resources (X. Li & Xia, 2022; Nie & Qiu, 2019). This provides a theoretical basis for testing an indirect path from role stress to emotional exhaustion. The ordering is theoretically plausible and can be tested statistically. It does not, however, show that the two states occurred at separate measured stages or at different times.

**Hypothesis 9a (H9a).**  *Role stress and emotional exhaustion jointly carry a serial indirect association between perceived algorithmic standardized guidance and work engagement.*

**Hypothesis 9b (H9b).**  *Role stress and emotional exhaustion jointly carry a serial indirect association between perceived algorithmic tracking evaluation and work engagement.*

**Hypothesis 9c (H9c).**  *Role stress and emotional exhaustion jointly carry a serial indirect association between perceived algorithmic behavioral constraints and work engagement.*

### 2.5. Role Stress, Emotional Exhaustion, and Work Engagement

Work engagement is a positive work-related state marked by vigor, dedication, and absorption (Schaufeli et al., 2006). Role ambiguity, conflict, and overload may weaken clarity and motivation, while emotional exhaustion reduces the energy available for sustained involvement. The S-S-O model therefore predicts that both forms of strain will be negatively associated with work engagement.

**Hypothesis 10 (H10).**  *Role stress is negatively associated with food-delivery workers’ work engagement.*

**Hypothesis 11 (H11).**  *Emotional exhaustion is negatively associated with food-delivery workers’ work engagement.*

### 2.6. Overall Social Support as a Boundary Condition

Overall social support encompasses the informational, emotional, and instrumental assistance that workers perceive within their social relationships (Cobb, 1976; T. P. Liang et al., 2011; Zhu et al., 2016). We focus on overall support rather than comparing informational and emotional support as separate moderators. As reported below, supplementary measurement analyses did not support a reliable distinction between the proposed item groups.

For standardized guidance, social support may partly replace the clarity and coping resources supplied by the algorithm. If workers already have substantial support, the additional strain-reducing association of standardized guidance may be weaker. This resource-substitution argument underlies H12 and H15.

For tracking evaluation and behavioral constraints, peer interaction may also make monitoring, rankings, penalties, and difficult experiences more salient. Higher overall support may therefore coincide with stronger positive associations between these control dimensions and strain. Stress sensitization and social comparison are possible explanations, but neither mechanism was measured or tested directly. The hypotheses concern moderation by overall support, not evidence of a sensitization process.

**Hypothesis 12 (H12).**  *Overall social support moderates the relationship between standardized guidance and emotional exhaustion, such that the negative association is weaker at higher levels of social support.*

**Hypothesis 13 (H13).**  *Overall social support moderates the relationship between tracking evaluation and emotional exhaustion, such that the positive association is stronger at higher levels of social support.*

**Hypothesis 14 (H14).**  *Overall social support moderates the relationship between behavioral constraints and emotional exhaustion, such that the positive association is stronger at higher levels of social support.*

**Hypothesis 15 (H15).**  *Overall social support moderates the relationship between standardized guidance and role stress, such that the negative association is weaker at higher levels of social support.*

**Hypothesis 16 (H16).**  *Overall social support moderates the relationship between tracking evaluation and role stress, such that the positive association is stronger at higher levels of social support.*

**Hypothesis 17 (H17).**  *Overall social support moderates the relationship between behavioral constraints and role stress, such that the positive association is stronger at higher levels of social support.*

**Figure 1:**
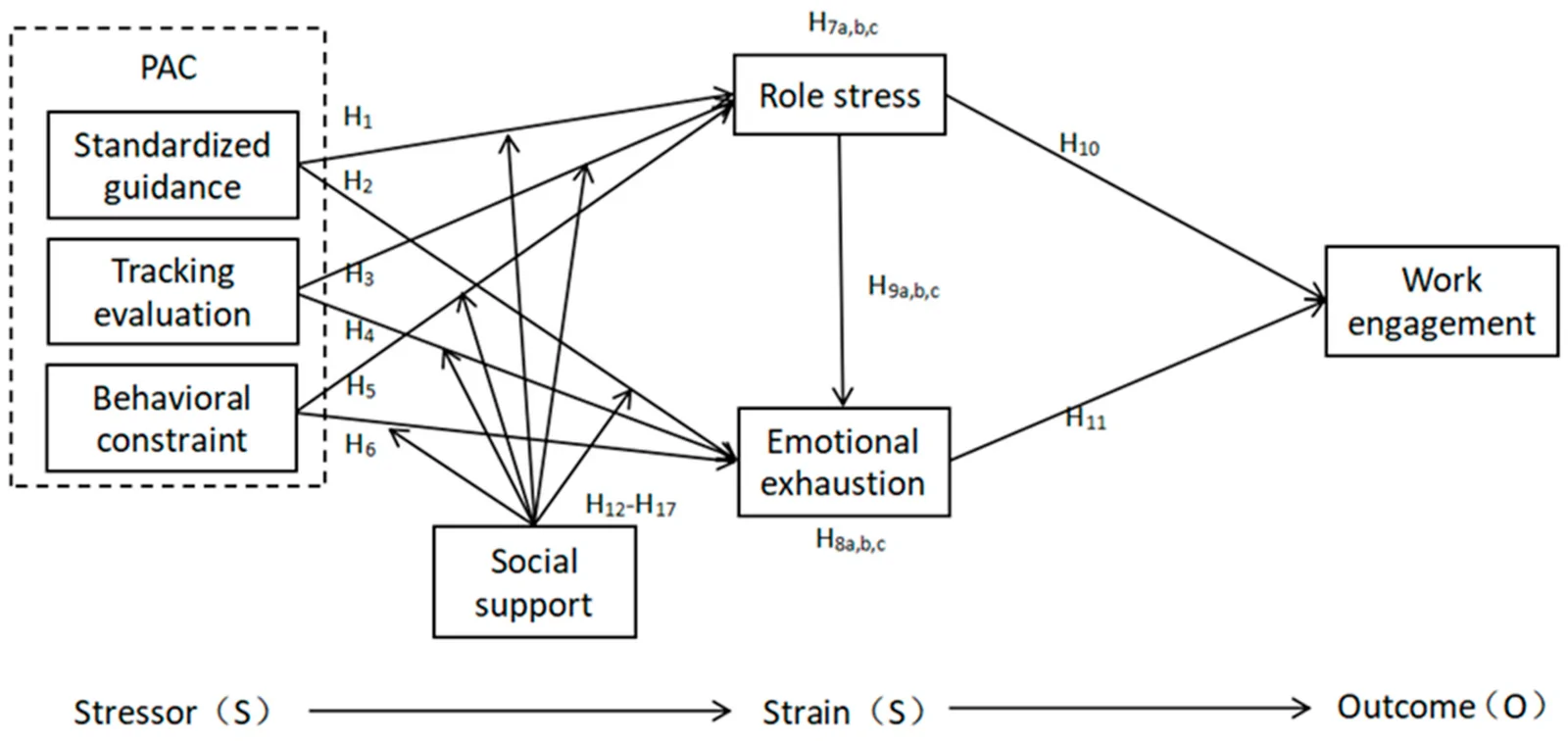
Proposed Theoretical Model.

The proposed model is presented in Figure 1.

## 3. Method

### 3.1. Sample and Data Collection

This cross-sectional study used single-source, self-report data. The study focused on platform gig work and sampled active food-delivery workers in China. Food-delivery work was chosen because algorithms continuously allocate orders, set route and time requirements, track location and progress, evaluate performance, and apply rewards and penalties. This makes food delivery a useful setting for studying perceived algorithmic control, although the sample is not statistically representative of all platform occupations. Questionnaire data were collected between January and June 2025 in two consecutive recruitment batches within the same cross-sectional survey. Both batches used the same questionnaire, target population, eligibility criteria, and measurement protocol. The two batches were combined as consecutive recruitment waves for the same study rather than treated as repeated observations of the same workers. Accordingly, the design was neither longitudinal nor time-lagged, and no temporal ordering is inferred from the two batches. Anonymous semi-structured interviews were used solely to provide contextual information; the interview records were neither linked to the questionnaire data nor included in the quantitative analyses. All respondents worked in immediate food delivery and used two-wheeled electric vehicles. The major platforms represented in the sample also used broadly similar core dispatch and delivery arrangements. Delivery mode and vehicle type therefore showed little meaningful variation and were not included as control variables.

Online participants were recruited through purposive and snowball procedures. The research team searched Douyin using food-delivery keywords, identified relevant worker discussion groups, and contacted potential participants through comments on worker-run social media accounts before sending private invitations. Eligible participants received an RMB 2 cash red envelope after completing the questionnaire. Only individuals who confirmed that they were currently working in food delivery could participate. The questionnaire system permitted only one completed submission per registered mobile phone number, thereby preventing multiple online submissions from the same number.

Offline recruitment combined personal referrals with field-site outreach. Food-delivery workers known to the researchers through relatives or friends were invited and asked to share the invitation with currently employed colleagues. Researchers also approached workers face to face during afternoon rest periods at common gathering places near the university and nearby commercial areas. Each participant was given a drink after completing the questionnaire. The designated field researchers used oral confirmation and the one-time incentive procedure to reduce duplicate participation. The same current-employment criterion was applied online and offline.

Responses were excluded if the respondent did not meet the current-worker criterion, completed the questionnaire in less than one minute, showed a clearly invariant response pattern, or failed an embedded consistency check. Because criterion-specific exclusion counts were not preserved, a detailed breakdown could not be reported. The mobile-number restriction prevented repeated online submissions using the same registered number, while oral confirmation and one-time incentive distribution were used offline. No comprehensive cross-channel identity log was retained because the survey was anonymous.

The first recruitment batch yielded 141 completed questionnaires, of which 119 were retained after screening. The second yielded 534 questionnaires, of which 489 were retained. Overall, 608 of the 675 questionnaires were included in the final analytic sample, with 67 excluded. Recruitment records preserved these aggregate batch totals, but the merged analytic dataset did not retain a case-level batch indicator; post hoc comparisons between batches were therefore not possible. Men accounted for 70.2% of the sample and women for 29.8%. The largest age groups were 31–40 years (32.6%) and 41–50 years (32.7%); 80.8% worked full time, and 70.4% had local household registration. The platform question allowed multiple responses: 521 respondents selected one platform category, 84 selected two categories, and 3 selected all three. This produced 698 platform selections in total and identified 87 respondents as multi-platform workers. Working tenure and monthly income varied across the sample. Because recruitment relied on purposive, convenience, and snowball channels rather than probability sampling, the sample should not be considered nationally representative. Table 1 summarizes the demographic and occupational characteristics.

### 3.2. Measures

All variables were measured with established five-point Likert scales (1 = strongly disagree, 5 = strongly agree) adapted to the food-delivery context. Before the formal survey, translated or contextually adapted items were reviewed for relevance and clarity. The final model included seven reflective constructs, with the indicators treated as manifestations of their underlying constructs; no formative measurement block was used. Higher-order specifications were considered only when the proposed lower-order dimensions could be assessed reliably, because both levels of measurement require explicit validation (Sarstedt et al., 2019). The complete wording and codes for all measurement items are provided in Appendix A (Table A1).

Standardized guidance (4 items) was measured using the perceived algorithmic control scale for gig workers developed by Pei et al. (2021). Representative items were: ‘The algorithm provides normative instructions for my work according to platform standards’; ‘The algorithm intelligently allocates my work tasks.’

Tracking evaluation (4 items) was also measured using Pei et al.’s (2021) scale. Representative items were: ‘The algorithm tracks and locates my geographical position in real time’; ‘The algorithm continuously follows up on my work progress.’

Behavioral constraints (3 items) were measured using the same scale (Pei et al., 2021). Representative items were: ‘The algorithm ranks my performance relative to other workers on the platform’; ‘The algorithm provides cash rewards at specific times to incentivize higher effort.’

Role stress was measured with 13 items adapted from C. Li and Zhang (2009), covering role conflict, role ambiguity, and role overload. The study focuses on overall role-related strain, and the three facets were extremely highly correlated in alternative specifications. All 13 items were therefore retained as reflective indicators of a single role stress construct. Representative items include: ‘I face conflicts between approaching order deadlines and compliance with traffic regulations’ and ‘I receive conflicting demands from the platform and customers when completing the same order.’

Emotional exhaustion was measured with five items from the scale revised by C. Li and Shi (2003). Representative items include: ‘Work leaves me feeling physically and emotionally depleted’ and ‘I feel drained at the end of each working day.’

Overall social support was measured with seven items adapted from T. P. Liang et al. (2011). The analysis focuses on overall perceived support rather than differences between informational and emotional support. Accordingly, all seven items were modeled as reflective indicators of one construct. Supplementary analyses did not reliably distinguish the proposed item groups in this sample. HTMT and the CFA latent correlation were approximately 1.001, and simultaneous subdimension models were unstable. A higher-order or two-factor specification would therefore create problems of discriminant validity and multicollinearity (Sarstedt et al., 2019). Representative items include: ‘When I encounter difficulties at work, there is someone who stands by me’ and ‘When I encounter difficulties at work, someone offers comfort and encouragement.’

Work engagement was measured with the nine-item Utrecht Work Engagement Scale (UWES-9) developed by Schaufeli et al. (2006). Although the scale covers vigor, dedication, and absorption, the present analysis treats work engagement as an overall construct. All nine items were therefore modeled as reflective indicators of one construct, while alternative dimensional models were examined as sensitivity checks. Representative items include: ‘I feel full of energy at work’ and ‘I am proud of the work that I do.’

### 3.3. Analytical Strategy

PLS-SEM was used for the primary structural analysis. This choice was not driven by formative measurement, because all retained constructs were reflective. PLS-SEM was appropriate because the model included six latent-score interaction terms, focused on explained variance in three endogenous constructs, and incorporated out-of-sample predictive assessment (Hair et al., 2019, 2022). We independently reproduced the model from the original 608-case dataset. The analysis used path weighting, reflective Mode A measurement, standardized two-stage interaction terms, and 5000 bootstrap resamples with a fixed seed. The moderation and simple-slope analyses followed established PLS guidance (Fassott et al., 2016). Direct and indirect associations were assessed with percentile bootstrap confidence intervals. Model evaluation included R^2^, adjusted R^2^, f^2^, inner VIF, reproduced-algorithm SRMR approximations, and repeated 10 × 10-fold construct-score cross-validation. Predictive assessment followed PLSpredict principles (Shmueli et al., 2019).

Robustness analyses added theoretically relevant controls in stages. The core set included occupational type, working tenure, average daily online duration, multi-platform work, gender, age, and income. The extended specification also included education, marital status, household registration, and platform indicators, with categorical variables examined through dummy coding. We compared the unadjusted and adjusted models rather than treating the controls as causal covariates.

Sensitivity to measurement and estimator choice was examined with covariance-based models. We estimated a seven-factor confirmatory factor analysis that matched the retained reflective specification using robust maximum likelihood. We also estimated an ordinal-item sensitivity model using WLSMV. We then estimated a CB-SEM model for the non-interaction main paths and indirect associations. Latent interactions remained in the PLS model because the CB-SEM analysis served as a robustness check rather than a replacement for the full moderated model. Common method variance was assessed in two ways. Harman’s single-factor analysis examined the variance explained by the first unrotated factor, and the full-collinearity approach examined VIF values for all construct scores. These diagnostics can identify serious common-method contamination, but they cannot establish its absence, especially because the questionnaire did not include a prespecified marker variable.

## 4. Results

### 4.1. Data Quality and Descriptive Statistics

The final analytic dataset contained 608 records with unique case identifiers. No values were missing for the 45 modeled indicators or the demographic and occupational variables used in the robustness analyses. All questionnaire indicators spanned the full 1–5 response range. Platform composition and the accounting of the multiple-response platform item are reported in Section 3.1 and Table 1.

Table 2 reports construct means, standard deviations, and bivariate correlations. Standardized guidance was negatively correlated with role stress (r = −0.334) and emotional exhaustion (r = −0.378), whereas tracking evaluation and behavioral constraints were positively correlated with both strain variables. Role stress and emotional exhaustion were positively correlated (r = 0.557), and both were negatively correlated with work engagement (r = −0.492 and −0.494, respectively). These correlations are descriptive associations and do not establish causal or temporal ordering.

### 4.2. Common Method Variance Diagnostics

Harman’s single-factor analysis showed that the first component accounted for 33.482% of the total variance, below the conventional 40% reference value. Full-collinearity VIFs for the seven construct scores ranged from 1.255 to 1.752, below 3.3. Neither diagnostic suggested serious common-method contamination (Kock, 2015; Podsakoff et al., 2003). However, the data were cross-sectional and came from the same respondents, and the questionnaire did not include a prespecified marker variable. Common method variance therefore cannot be ruled out.

### 4.3. Measurement Model

All 45 retained indicators were modeled reflectively. Standardized loadings ranged from 0.743 to 0.856, all above 0.70. Cronbach’s alpha ranged from 0.801 to 0.955, composite reliability from 0.883 to 0.960, and AVE from 0.596 to 0.715. Collectively, these results support indicator reliability, internal consistency, and convergent validity (Hair et al., 2019, 2022). Table 3 reports the indicator- and construct-level measurement results.

**Table 3:** Reflective Measurement Model: Indicator Loadings and Convergent Validity.

Construct	Item	Factor Loading	Cronbach’s α	CR	AVE
A	A1	0.821	0.827	0.885	0.659
	A2	0.789			
	A3	0.823			
	A4	0.814			
B	B1	0.822	0.851	0.899	0.69
	B2	0.837			
	B3	0.807			
	B4	0.856			
C	C1	0.844	0.801	0.883	0.715
	C2	0.846			
	C3	0.847			
RS	RC1	0.796	0.955	0.96	0.648
	RC2	0.799			
	RC3	0.795			
	RA1	0.795			
	RA2	0.813			
	RA3	0.791			
	RA4	0.808			
	RA5	0.823			
	RO1	0.814			
	RO2	0.803			
	RO3	0.8			
	RO4	0.803			
	RO5	0.823			
EE	E1	0.837	0.894	0.922	0.703
	E2	0.845			
	E3	0.822			
	E4	0.848			
	E5	0.838			
SS	EMS1	0.824	0.917	0.934	0.668
	EMS2	0.808			
	EMS3	0.819			
	EMS4	0.807			
	IFS1	0.838			
	IFS2	0.809			
	IFS3	0.815			
WE	Y1	0.782	0.915	0.93	0.596
	Y2	0.779			
	Y3	0.781			
	Y4	0.743			
	Y5	0.768			
	Y6	0.776			
	Y7	0.781			
	Y8	0.758			
	Y9	0.781			

Note: All constructs were modeled reflectively. α = Cronbach’s alpha; CR = composite reliability; AVE = average variance extracted.

### 4.4. Discriminant Validity

For all seven constructs, AVE exceeded maximum shared variance (MSV), and MaxR(H) ranged from 0.883 to 0.960 (Table 4). The largest HTMT value in the retained seven-construct model was 0.603, below the conservative threshold of 0.85 (Table 5) (Fornell & Larcker, 1981; Henseler et al., 2015). These findings support discriminant validity for the retained construct-level model. We did not interpret the informational and emotional social support item groups as separate constructs. Supplementary diagnostics yielded HTMT and CFA latent correlations of approximately 1.001; therefore, the analysis retains overall social support.

**Table 4:** Construct-Level Reliability and Validity.

Construct	Items	α	CR	AVE	MSV	MaxR(H)
A	4	0.827	0.885	0.659	0.143	0.886
B	4	0.851	0.899	0.690	0.134	0.900
C	3	0.801	0.883	0.715	0.139	0.883
RS	13	0.955	0.960	0.648	0.311	0.960
EE	5	0.894	0.922	0.703	0.311	0.922
SS	7	0.917	0.934	0.668	0.165	0.934
WE	9	0.915	0.930	0.596	0.245	0.930

Note: AVE exceeded MSV for every construct. MaxR(H) = maximal reliability.

**Table 5:** Heterotrait–Monotrait Ratios (HTMT).

Construct	A	B	C	RS	EE	SS	WE
A	—	—	—	—	—	—	—
B	0.318	—	—	—	—	—	—
C	0.319	0.332	—	—	—	—	—
RS	0.375	0.347	0.426	—	—	—	—
EE	0.439	0.382	0.417	0.603	—	—	—
SS	0.349	0.414	0.303	0.428	0.447	—	—
WE	0.414	0.224	0.368	0.527	0.546	0.396	—

Note: The maximum off-diagonal HTMT value was 0.603. Em dashes indicate values not reported on the diagonal or in the unrepeated upper triangle.

### 4.5. Structural Model Quality

The primary PLS model explained 32.7% of the variance in role stress, 43.0% in emotional exhaustion, and 31.3% in work engagement. Inner VIF values ranged from 1.108 to 1.485, with no indication of problematic structural collinearity. Most f^2^ values were negligible or small. The largest was 0.123 for the association between role stress and emotional exhaustion, followed by values of approximately 0.100–0.102 for the associations of role stress and emotional exhaustion with work engagement. Repeated 10 × 10-fold construct-score cross-validation produced positive Q^2^ predict values for all endogenous constructs (Shmueli et al., 2019). The reproduced-algorithm SRMR approximations were 0.031 for the saturated measurement model and 0.039 for the estimated structural model. These values are independent reproduction diagnostics rather than proprietary SmartPLS output.

### 4.6. Direct and Lower-Order Effects

Standardized guidance was negatively associated with role stress (β = −0.132, *p* < 0.001) and emotional exhaustion (β = −0.138, *p* < 0.001), supporting H1 and H2. Tracking evaluation was positively associated with role stress (β = 0.126, *p* = 0.001) and emotional exhaustion (β = 0.101, *p* = 0.004), supporting H3 and H4. Behavioral constraints were positively associated with role stress (β = 0.207, *p* < 0.001) and emotional exhaustion (β = 0.095, *p* = 0.007), supporting H5 and H6. Role stress was positively associated with emotional exhaustion (β = 0.323, *p* < 0.001). Role stress and emotional exhaustion were negatively associated with work engagement (β = −0.316 and −0.318, both *p* < 0.001), supporting H10 and H11. The required lower-order effects of overall social support were also significant and positive for role stress (β = 0.215) and emotional exhaustion (β = 0.136). Table 6 reports the corresponding explained-variance statistics, and Table 7 reports the standardized coefficients, bootstrap confidence intervals, effect sizes, collinearity diagnostics, and hypothesis decisions.

**Table 6:** Explanatory and Predictive Assessment.

Endogenous Construct	R^2^	Adjusted R^2^	RMSE	MAE	Q^2^ Predict	Assessment
RS	0.327	0.319	0.833	0.687	0.310	Positive
EE	0.430	0.423	0.811	0.674	0.345	Positive
WE	0.313	0.311	0.882	0.742	0.225	Positive

Note: Predictive values are based on repeated 10 × 10-fold construct-score cross-validation and are not the proprietary indicator-level PLSpredict output.

**Table 7:** Direct and Lower-Order Structural Associations.

Hypothesis	Path	β	SE	*p*	95% CI	f^2^/VIF	Decision
H1	A → RS	−0.132	0.036	<0.001	[−0.203, −0.060]	0.022/1.205	Supported
H3	B → RS	0.126	0.039	0.001	[0.050, 0.203]	0.018/1.305	Supported
H5	C → RS	0.207	0.039	<0.001	[0.133, 0.282]	0.052/1.223	Supported
Additional main effect	SS → RS	0.215	0.040	<0.001	[0.137, 0.291]	0.053/1.289	Lower-order path
H2	A → EE	−0.138	0.035	<0.001	[−0.207, −0.071]	0.027/1.231	Supported
H4	B → EE	0.101	0.035	0.004	[0.032, 0.173]	0.014/1.329	Supported
H6	C → EE	0.095	0.035	0.007	[0.026, 0.164]	0.012/1.287	Supported
Additional main effect	SS → EE	0.136	0.037	<0.001	[0.065, 0.210]	0.024/1.358	Lower-order path
Additional main effect	RS → EE	0.323	0.038	<0.001	[0.246, 0.396]	0.123/1.485	Lower-order path
H10	RS → WE	−0.316	0.037	<0.001	[−0.388, −0.241]	0.100/1.451	Supported
H11	EE → WE	−0.318	0.038	<0.001	[−0.393, −0.244]	0.102/1.451	Supported

Note: Confidence intervals are percentile bootstrap intervals from 5000 resamples. Social-support and RS → EE coefficients are lower-order paths required for interpreting the moderated model.

### 4.7. Indirect and Serial Indirect Associations

The indirect associations through role stress and emotional exhaustion were statistically significant for all three dimensions of perceived algorithmic control. The serial estimates through role stress and then emotional exhaustion were 0.014 for standardized guidance, −0.013 for tracking evaluation, and −0.021 for behavioral constraints. All three bootstrap confidence intervals excluded zero. H7a–H8c and H9a–H9c were therefore supported statistically. Although significant, the serial estimates were small in absolute magnitude. They are best interpreted as theory-consistent serial indirect associations, not as strong evidence of a two-stage process unfolding over time. Table 8 summarizes the indirect, serial indirect, and total indirect associations.

**Table 8:** Indirect, Serial Indirect, and Total Indirect Associations.

Hypothesis	Indirect Path	Estimate	SE	*p*	95% CI	Decision
H7a	A → RS → WE	0.042	0.013	0.001	[0.018, 0.069]	Supported
H8a	A → EE → WE	0.044	0.013	0.001	[0.021, 0.072]	Supported
H9a	A → RS → EE → WE	0.014	0.004	0.002	[0.006, 0.023]	Supported
Total indirect	A → WE (total indirect)	0.099	0.020	<0.001	[0.061, 0.142]	Significant total indirect
H7b	B → RS → WE	−0.040	0.013	0.002	[−0.066, −0.015]	Supported
H8b	B → EE → WE	−0.032	0.012	0.007	[−0.057, −0.010]	Supported
H9b	B → RS → EE → WE	−0.013	0.005	0.004	[−0.023, −0.005]	Supported
Total indirect	B → WE (total indirect)	−0.085	0.020	<0.001	[−0.126, −0.045]	Significant total indirect
H7c	C → RS → WE	−0.065	0.015	<0.001	[−0.096, −0.039]	Supported
H8c	C → EE → WE	−0.030	0.012	0.011	[−0.054, −0.008]	Supported
H9c	C → RS → EE → WE	−0.021	0.006	<0.001	[−0.034, −0.012]	Supported
Total indirect	C → WE (total indirect)	−0.117	0.021	<0.001	[−0.159, −0.077]	Significant total indirect

Note: Serial estimates (H9a–H9c) are small. Statistical significance does not establish temporal ordering in cross-sectional data.

### 4.8. Moderation, Simple Slopes, and Interaction Plots

**Figure 2:**
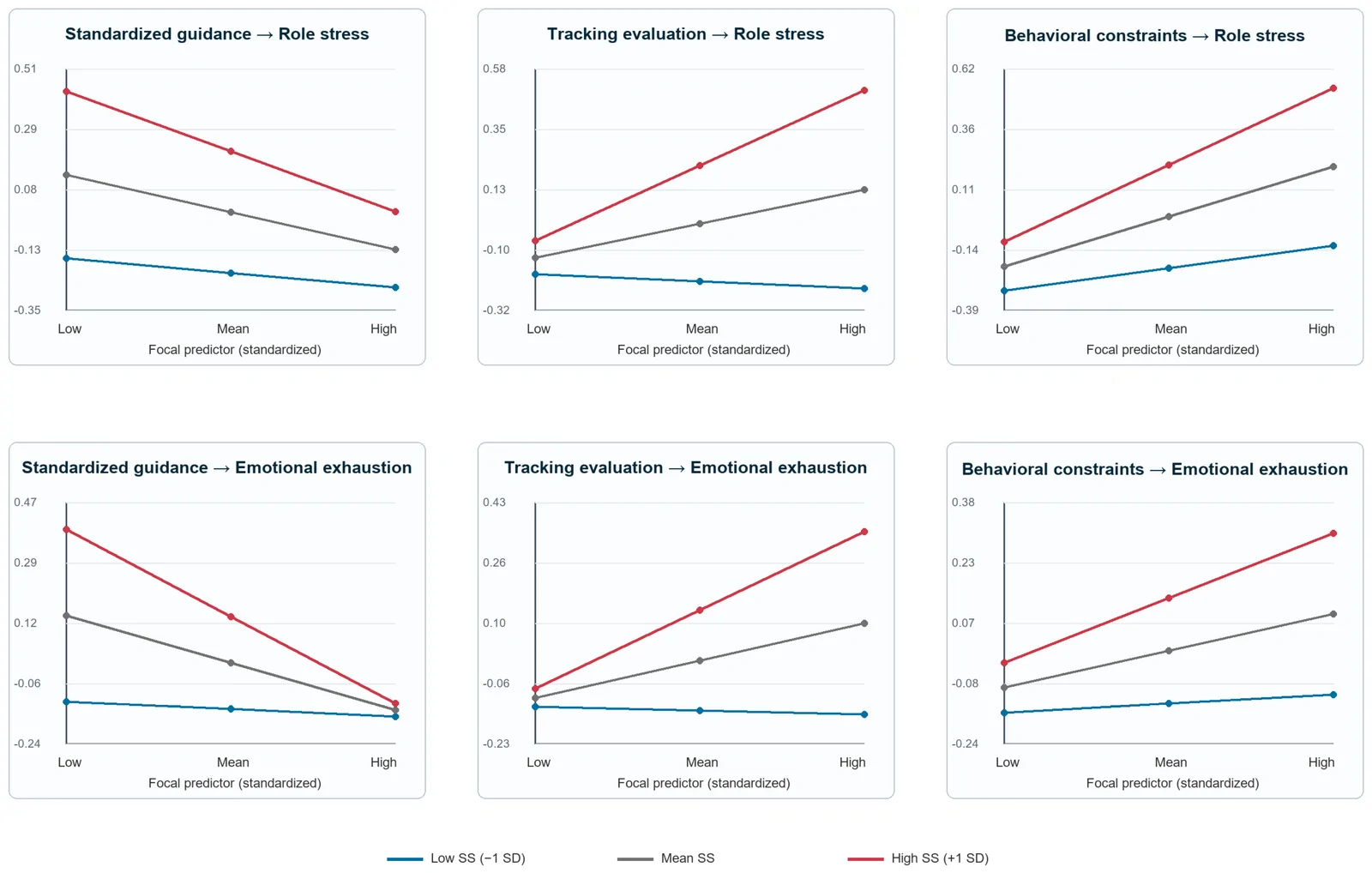
Simple-Slope Interaction Plots for Overall Social Support.

The interaction coefficients for standardized guidance were negative for role stress (β = −0.077, *p* = 0.028) and for emotional exhaustion (β = −0.111, *p* < 0.001). The simple slopes became more negative as social support increased. H12 and H15 predicted the opposite pattern, so neither hypothesis was supported. The interaction coefficients for tracking evaluation were positive for role stress (β = 0.144, *p* < 0.001) and for emotional exhaustion (β = 0.104, *p* = 0.001), supporting H16 and H13. The interaction coefficients involving behavioral constraints were also positive for role stress (β = 0.109, *p* = 0.003) and for emotional exhaustion (β = 0.069, *p* = 0.032), supporting H17 and H14. Interaction f^2^ values ranged from 0.007 to 0.027, indicating negligible-to-small incremental effects, and all interaction-model VIFs were below 1.18. Resource complementarity, rather than substitution, may explain the unsupported H12/H15 pattern. Supportive contacts may help workers understand and apply standardized guidance, making its negative association with strain stronger when support is high. This post hoc explanation was not tested directly. Figure 2 shows the corresponding simple slopes. Table 9 summarizes the interaction coefficients and simple slopes.

**Table 9:** Interaction Coefficients and Simple Slopes.

Hypothesis	Interaction	β (*p*)	95% CI	Low SS	Mean SS	High SS	Decision
H15	A × SS → RS	−0.077 (0.028)	[−0.145, −0.006]	−0.051	−0.132	−0.213	Not supported; opposite direction
H16	B × SS → RS	0.144 (<0.001)	[0.077, 0.213]	−0.027	0.126	0.280	Supported
H17	C × SS → RS	0.109 (0.003)	[0.040, 0.182]	0.094	0.207	0.321	Supported
H12	A × SS → EE	−0.111 (<0.001)	[−0.175, −0.044]	−0.022	−0.138	−0.255	Not supported; opposite direction
H13	B × SS → EE	0.104 (<0.001)	[0.042, 0.165]	−0.010	0.101	0.212	Supported
H14	C × SS → EE	0.069 (0.032)	[0.005, 0.132]	0.023	0.095	0.166	Supported

Note: Simple slopes are evaluated at −1 SD, the mean, and +1 SD of overall social support. For standardized guidance, higher support produced a larger negative slope, contrary to H12 and H15.

### 4.9. Control-Variable Robustness

The main-effect directions and the moderation patterns for tracking evaluation and behavioral constraints were stable across the unadjusted, core-control, extended-control, and fully dummy-coded models. Core controls included occupational type, working tenure, average daily online duration, multi-platform work, gender, age, and income; the extended models added education, marital status, household registration, and platform indicators. The interaction between standardized guidance and social support for role stress was the only focal interaction that was not significant in every specification. Its coefficient changed from β = −0.077 (*p* = 0.028) without controls to β = −0.065 (*p* = 0.074) in the fully dummy-coded model. This result should therefore be treated as sensitive to the control specification. Table 10 summarizes the control-variable robustness results.

**Table 10:** Control-Variable Robustness Summary.

Specification	Outcome	R^2^	Term 1: β (*p*)	Term 2: β (*p*)	Term 3: β (*p*)
No controls	RS	0.327	A × SS → RS: −0.077 (0.028)	B × SS → RS: 0.144 (<0.001)	C × SS → RS: 0.109 (0.004)
No controls	EE	0.430	A × SS → EE: −0.111 (<0.001)	B × SS → EE: 0.104 (<0.001)	C × SS → EE: 0.069 (0.032)
No controls	WE	0.313	RS → WE: −0.316 (<0.001)	EE → WE: −0.318 (<0.001)	—
Core controls	RS	0.334	A × SS → RS: −0.081 (0.021)	B × SS → RS: 0.140 (<0.001)	C × SS → RS: 0.109 (0.004)
Core controls	EE	0.440	A × SS → EE: −0.113 (<0.001)	B × SS → EE: 0.101 (0.002)	C × SS → EE: 0.071 (0.028)
Core controls	WE	0.318	RS → WE: −0.318 (<0.001)	EE → WE: −0.314 (<0.001)	—
Extended controls	RS	0.344	A × SS → RS: −0.075 (0.035)	B × SS → RS: 0.142 (<0.001)	C × SS → RS: 0.119 (0.002)
Extended controls	EE	0.442	A × SS → EE: −0.113 (0.001)	B × SS → EE: 0.101 (0.002)	C × SS → EE: 0.073 (0.029)
Extended controls	WE	0.322	RS → WE: −0.321 (<0.001)	EE → WE: −0.313 (<0.001)	—
Dummy coded controls	RS	0.360	A × SS → RS: −0.065 (0.074)	B × SS → RS: 0.136 (<0.001)	C × SS → RS: 0.118 (0.003)
Dummy coded controls	EE	0.450	A × SS → EE: −0.110 (0.003)	B × SS → EE: 0.105 (0.002)	C × SS → EE: 0.077 (0.027)
Dummy coded controls	WE	0.354	RS → WE: −0.316 (<0.001)	EE → WE: −0.314 (<0.001)	—

Note: RS and EE rows report the three interaction terms; WE rows report the RS and EE predictors. Complete control-model output is retained in the analysis archive.

### 4.10. CB-SEM and Predictive Robustness

The seven-factor MLR CFA showed good fit (robust CFI = 0.999, robust TLI = 0.999, robust RMSEA = 0.006, SRMR = 0.025). The MLR CB-SEM model, which excluded latent interactions, also fitted the data well, χ^2^(928) = 996.83, *p* = 0.058, CFI = 0.996, TLI = 0.995, RMSEA = 0.011, SRMR = 0.032. The directions and significance decisions for the non-interaction paths were consistent with the PLS findings, as were the signs and significance of the indirect associations. CB-SEM was used to assess sensitivity to estimator and measurement-model choice, not to replace the six-interaction PLS model. Together with the positive Q^2^ predict values in Table 6, these results suggest that the central non-interaction findings do not depend on a single estimator. The moderated paths, however, remain subject to the sensitivity qualifications noted above. Table 11 compares the non-interaction PLS and CB-SEM path estimates.

**Table 11:** PLS and CB-SEM Comparison for Non-Interaction Paths.

Path	PLS β	CB-SEM β	CB-SEM *p*	Direction	Conclusion
A → RS	−0.132	−0.178	<0.001	Same	Consistent
B → RS	0.126	0.103	0.030	Same	Consistent
C → RS	0.207	0.263	<0.001	Same	Consistent
SS → RS	0.215	0.245	<0.001	Same	Consistent
A → EE	−0.138	−0.180	<0.001	Same	Consistent
B → EE	0.101	0.086	0.039	Same	Consistent
C → EE	0.095	0.125	0.006	Same	Consistent
SS → EE	0.136	0.151	<0.001	Same	Consistent
RS → EE	0.323	0.387	<0.001	Same	Consistent
RS → WE	−0.316	−0.304	<0.001	Same	Consistent
EE → WE	−0.318	−0.370	<0.001	Same	Consistent

Note: CB-SEM coefficients are completely standardized estimates. Latent interactions were excluded from the CB-SEM sensitivity model.

## 5. Conclusions and Discussion

### 5.1. Discussion of Principal Findings

#### 5.1.1. Dimension-Specific Associations of Algorithmic Control

First, the three dimensions of perceived algorithmic control were associated with strain in different directions. Standardized guidance was negatively associated with role stress and emotional exhaustion, whereas tracking evaluation and behavioral constraints were positively associated with both; behavioral constraints showed the strongest association with role stress. From a JD-R perspective, standardized guidance may function as a resource because it clarifies tasks, routes, and performance expectations. Tracking evaluation and behavioral constraints may function as demands because they increase visibility, time pressure, accountability, and restrictions on discretion. The results therefore support examining the functions of established control dimensions separately rather than assuming that algorithmic control has a uniformly beneficial or harmful association.

Several alternative explanations remain possible. More experienced, algorithmically literate, or successful workers may view guidance more favorably and also report less strain. Conversely, workers who are already under stress may pay more attention to monitoring and penalties. Platform practices, local delivery density, income dependence, and other unmeasured worker characteristics may also shape these perceptions. Because all variables were measured at the same time, the observed directions are theory-guided associations rather than evidence that an algorithmic feature caused later strain. The findings are relevant to platform gig work. However, the evidence comes specifically from Chinese food-delivery workers and reflects their perceptions of algorithmic management rather than objective platform records.

#### 5.1.2. Independent and Serial Indirect Associations

Second, role stress and emotional exhaustion each carried significant indirect associations between the three dimensions of algorithmic control and work engagement. This pattern fits the S-S-O structure while keeping role-related strain conceptually distinct from emotional-energy depletion. Difficulty understanding, reconciling, or meeting role expectations may reduce attention and motivation, whereas emotional exhaustion may leave workers with less energy for vigor and dedication. Considering both variables therefore reveals two related pathways between perceived algorithmic work conditions and engagement.

The serial indirect estimates through role stress and emotional exhaustion were statistically significant but small in absolute magnitude (approximately 0.013–0.021). Small serial estimates are unsurprising because they multiply several modest component paths, while parallel indirect paths account for part of the overall association. They may also indicate that role stress is only one of several antecedents of emotional exhaustion and that much of the association with engagement does not pass through the full chain. Scale overlap, omitted variables, and the concurrent measurement of all variables offer other possible explanations for the statistical significance. The serial findings should therefore be read as small, theory-consistent indirect associations, not as evidence of a strong psychological process unfolding in two temporal stages.

#### 5.1.3. Overall Social Support and the Unsupported H12/H15 Predictions

Third, overall social support did not act as a uniform buffer against strain. Higher support was associated with steeper positive slopes for tracking evaluation and behavioral constraints in relation to role stress and emotional exhaustion. Workers’ networks may circulate accounts of monitoring, rankings, penalties, and difficult delivery experiences, making algorithmic demands more salient or encouraging unfavorable social comparison. In this sense, support may coexist with stress sensitization rather than simply offset demands. Neither sensitization nor social comparison was measured, however, and workers may also seek more support when they are already under greater stress. These interpretations remain tentative rather than demonstrated mechanisms.

H12 and H15 predicted that social support would weaken the negative associations of standardized guidance with emotional exhaustion and role stress, as expected under resource substitution. Instead, these associations became more negative at higher levels of support. Resource complementarity is one possible explanation. Supportive peers may help workers understand or apply algorithmic guidance, making that guidance more useful rather than redundant. Selection is another possibility, because workers in stronger networks may differ in experience, strategy sharing, or familiarity with platform rules. The interaction effects were negligible to small, and the interaction between standardized guidance and social support for role stress was no longer conventionally significant in the fully dummy-coded model. Accordingly, H12 and H15 remain unsupported. The opposite-direction pattern is informative, but it should not be treated as evidence of a new mechanism.

The supplementary measurement analyses also explain why the study treats social support as one overall construct rather than separating informational and emotional support. The proposed item groups were almost perfectly correlated in this sample (HTMT and the CFA latent correlation were approximately 1.001), and the simultaneous subdimension models were unstable. Food-delivery workers may experience different forms of assistance as a single support resource, or the adapted items may not be specific enough to distinguish them. Retaining one construct avoids artificial multicollinearity, but it does not mean that the subdimensions are theoretically identical. The moderation results therefore apply only to overall perceived support.

#### 5.1.4. Robustness, Sensitive Paths, and Estimator Consistency

Most focal paths were stable across the unadjusted and control-variable specifications. The exception was the interaction between standardized guidance and overall social support for role stress: its *p* value changed from 0.028 without controls to 0.074 in the fully dummy-coded model. This sensitivity suggests that the estimate depends partly on how demographic, occupational, and platform differences are represented. It should therefore be treated as tentative and specification-dependent rather than as a robust boundary condition. The remaining main paths and most interaction directions were stable, but that stability cannot rule out omitted-variable bias.

The non-interaction PLS and CB-SEM models yielded consistent coefficient directions and significance decisions, and the CFA fitted the data well. This convergence suggests that the central non-interaction pattern is not simply an artifact of a single estimator or implementation of the measurement model. Positive Q^2^ predict values also indicate that the reproduced PLS model provides predictive information about the endogenous construct scores. Even so, CB-SEM did not estimate the six latent interactions, agreement across estimators cannot establish causality, and neither approach resolves the common-method, sampling, or measurement limitations. The robustness checks therefore increase confidence in the non-interaction pattern, while the moderation and causal claims remain appropriately limited.

### 5.2. Theoretical Contributions

First, the study compares three established dimensions of perceived algorithmic control within one S-S-O model. The opposite directions observed for standardized guidance versus tracking evaluation and behavioral constraints show how an aggregate measure can conceal resource-like and demand-like functions. The contribution is the direct comparison of these dimensions, not a claim to have introduced the three-dimensional conceptualization.

Second, the study jointly considers role stress and emotional exhaustion in relation to work engagement. Their independent indirect associations suggest that role-related difficulties and emotional depletion provide distinct but connected explanations of how algorithmic work conditions relate to engagement. The small serial estimates are consistent with the proposed ordering, but they do not establish temporal stages.

Third, the moderation results qualify the common assumption that social support is always protective. Overall support coincided with stronger associations involving tracking evaluation and behavioral constraints and did not produce the expected resource-substitution pattern for standardized guidance. This result points to a boundary of stress-buffering arguments in algorithmically managed work. Stress sensitization, social comparison, and resource complementarity remain possibilities for future direct tests.

Fourth, the study provides context-specific evidence from food-delivery workers in China. In this setting, platform coordination, monitoring, and performance rules are especially visible. Food delivery is therefore useful for identifying dimension-specific and support-contingent patterns. The findings are not, however, assumed to be universal across platform occupations, platforms, or regulatory settings.

### 5.3. Practical Implications

Because the evidence is cross-sectional and based on workers’ perceptions, the following implications should be understood as possibilities for platform experimentation and evaluation, not as established causal prescriptions.

First, the negative associations of standardized guidance with role stress and emotional exhaustion suggest that platforms might improve the clarity, consistency, and contextual usefulness of navigation, task-allocation, and work-instruction functions. Alternative guidance designs could be piloted and evaluated in terms of worker-reported strain, delivery quality, and safety before wider use. The present results do not show that simply increasing guidance will reduce strain.

Second, platforms might review the intensity, transparency, and contestability of monitoring, rankings, rewards, and penalties. Possible steps include explaining performance rules more clearly, providing channels for correcting algorithmic errors, allowing context-sensitive exceptions for events outside workers’ control, and consulting workers when rules are revised. Any changes should be evaluated prospectively rather than assumed to improve psychological outcomes.

Third, peer-support or assistance programs could complement, rather than replace, structural reviews of algorithmic demands. Because greater overall support was associated with steeper slopes in several models, such programs may need safeguards against rumor amplification, punitive comparison, or repeated circulation of stressful incidents. Better information quality, access to formal problem-resolution channels, and regular checks for unintended effects may be more useful than assuming that more peer interaction is automatically protective.

Finally, because the interaction between standardized guidance and overall social support was sensitive to the control specification, platforms and policymakers should not act on a single moderation coefficient. Staged trials, preregistered evaluation criteria, and repeated measurement could show whether apparently beneficial or adverse patterns recur across worker groups, platforms, and operating conditions.

### 5.4. Limitations and Future Research

First, the study is cross-sectional. The proposed paths represent theory-guided statistical associations and cannot establish either causal direction or the temporal ordering of role stress, emotional exhaustion, and work engagement. Future studies could use time-lagged panels, experience sampling, natural experiments, or randomized platform interventions where feasible.

Second, all focal constructs were reported by the same respondents. Harman’s test and full-collinearity VIFs did not indicate serious common-method bias, but the questionnaire did not include a prespecified marker variable. Common method variance, consistency motifs, and transient affect therefore cannot be ruled out. Future surveys could include marker variables, temporally separated measurements, supervisor or platform records, and objective behavioral indicators.

Third, purposive, convenience, and snowball recruitment limit representativeness. Food delivery is a typical and algorithmically intensive form of platform gig work. Nevertheless, the sample is not a probability sample of all Chinese food-delivery workers, and the findings should not be generalized automatically to other platform occupations. Replication is needed across regions, forms of platform work, regulatory settings, and probability-based sampling frames.

Fourth, recruitment records preserved the aggregate questionnaire totals for each batch, but the merged analytic file did not retain a case-level batch label or criterion-specific exclusion counts. The online questionnaire permitted only one submission per registered mobile phone number, whereas duplicate control in the offline survey relied on oral confirmation and a one-time incentive. The anonymous survey did not retain a comprehensive cross-channel identity log. Therefore, the criterion-level screening process and duplicate control across channels cannot be reconstructed independently. Future studies should preserve a preregistered exclusion log, a non-identifying case-level batch indicator, and privacy-preserving records for cross-channel duplicate control.

Fifth, the informational and emotional social support item groups could not be distinguished reliably in this sample. Treating support as one overall construct means that the study cannot determine whether particular forms of support buffer, amplify, or complement specific algorithmic conditions. Future work should develop context-specific items, validate them qualitatively with workers, and test multidimensional support measures in independent samples before comparing subdimension effects.

Sixth, stress sensitization, social comparison, resource substitution, and resource complementarity were not measured directly. They represent possible interpretations of the moderation pattern rather than tested mediating mechanisms. Future studies should measure these processes explicitly and use longitudinal moderated-mediation or experimental designs to distinguish among them.

Finally, the interaction effects were generally negligible to small. One focal interaction was sensitive to the control specification, and the CB-SEM analysis did not include latent interactions. Replication should therefore emphasize confidence intervals, preregistered control sets, alternative estimators that can model interactions, and out-of-sample confirmation rather than relying only on binary significance decisions.

## Figures and Tables

**Table 1:** Demographic and Occupational Characteristics of the Sample (N = 608).

Variables	Type	Sample Size	Percent of Respondents
Occupational Type	Part-time	117	19.20%
	Full-time	491	80.80%
Working Tenure (a)	a < 3 months	54	8.90%
	3 months ≤ a < 6 months	66	10.90%
	6 months ≤ a < 1 year	138	22.70%
	1 year ≤ a < 3 years	142	23.40%
	3 years ≤ a < 5 years	144	23.70%
	5 years ≤ a	64	10.50%
Average Daily Online Duration (b)	b < 6 h	72	11.80%
	6 h ≤ b < 8 h	284	46.70%
	8 h ≤ b < 10 h	167	27.50%
	10 h ≤ b	85	14.00%
Working Platform	Meituan	408	67.1%
	Eleme	275	45.2%
	Others	15	2.5%
Gender	Male	427	70.20%
	Female	181	29.80%
Age	16–20 years old	56	9.20%
	21–30 years old	107	17.60%
	31–40 years old	198	32.60%
	41–50 years old	199	32.70%
	51 years old and above	48	7.90%
Level of education	Senior High School and Below	221	36.30%
	Junior College	216	35.50%
	Bachelor’s Degree	104	17.10%
	Postgraduate Degree	67	11.00%
Marital status	Unmarried	130	21.40%
	Married	435	71.50%
	Others	43	7.10%
Work Income	≤2000 Yuan	52	8.60%
	2001–4000 Yuan	81	13.30%
	4001–6000 Yuan	174	28.60%
	6001–8000 Yuan	156	25.70%
	8001–10,000 Yuan	71	11.70%
	>10,000 Yuan	74	12.20%
Household Registration	Local	428	70.40%
	Non-local	180	29.60%

Note: Working platform was a multiple-response item. The 608 respondents made 698 platform selections: 521 selected one platform category, 84 selected two categories, and 3 selected all three categories. Thus, 87 respondents worked across multiple platform categories. Percentages for Meituan, Eleme, and other platforms use N = 608 as the denominator and sum to more than 100%.

**Table 2:** Descriptive Statistics and Bivariate Correlations (N = 608).

Component	A	B	C	RS	EE	SS	WE
A	1						
B	−0.266 **	1					
C	−0.260 **	0.272 **	1				
RS	−0.334 **	0.311 **	0.372 **	1			
EE	−0.378 **	0.331 **	0.353 **	0.557 **	1		
SS	−0.304 **	0.362 **	0.258 **	0.399 **	0.404 **	1	
WE	0.361 **	−0.195 **	−0.314 **	−0.492 **	−0.494 **	−0.362 **	1
Mean (M)	2.69	3.4	3.33	3.23	3.18	3.26	2.66
Standard Deviation (SD)	0.91	0.95	0.94	0.96	1.02	0.94	0.87

Note: ** *p* < 0.01. A = standardized guidance; B = tracking evaluation; C = behavioral constraints; RS = role stress; EE = emotional exhaustion; SS = overall social support; WE = work engagement. These abbreviations are used consistently in Table 2, Table 3, Table 4, Table 5, Table 6, Table 7, Table 8, Table 9, Table 10 and Table 11.

## Data Availability

The de-identified data are not publicly posted because the consent process did not specify unrestricted public archiving. Subject to institutional requirements and appropriate privacy safeguards, data may be made available by the corresponding author upon reasonable request.

## Appendix A

Table A1 provides the full English wording of the 45 adapted measurement items used in the analysis, together with their item codes and construct assignments.

**Table A1:** Full List of Measurement Items.

Construct	Code	Measurement Item
Standardized guidance	A1	The algorithm intelligently allocates my work tasks.
Standardized guidance	A2	The algorithm provides normative instructions for my work according to platform standards.
Standardized guidance	A3	The algorithm provides substantial information that helps me complete my work tasks.
Standardized guidance	A4	The algorithm provides real-time feedback about my work performance.
Tracking evaluation	B1	The algorithm tracks and locates my geographical position in real time.
Tracking evaluation	B2	The algorithm continuously follows my work progress.
Tracking evaluation	B3	The algorithm monitors my work attitude in real time.
Tracking evaluation	B4	The algorithm automatically evaluates the quality of my delivery service.
Behavioral constraints	C1	The algorithm ranks my performance relative to other workers on the platform.
Behavioral constraints	C2	The algorithm provides cash rewards at specific times to encourage greater effort.
Behavioral constraints	C3	The algorithm imposes a fine when my work does not meet platform requirements.
Role stress	RC1	I face conflicts between approaching order deadlines and compliance with traffic regulations.
Role stress	RC2	I receive conflicting demands from the platform and customers when completing the same order.
Role stress	RC3	I must deal with different work situations and handle similar tasks in different ways.
Role stress	RA1	The goals and objectives of my delivery work are unclear.
Role stress	RA2	I am unsure what the platform expects of me.
Role stress	RA3	I am unsure about my responsibilities as a food-delivery worker.
Role stress	RA4	I am unclear about the extent of my responsibilities.
Role stress	RA5	My work responsibilities are not clearly defined.
Role stress	RO1	I need part of my workload to be reduced.
Role stress	RO2	I feel overloaded at work.
Role stress	RO3	I have too many responsibilities.
Role stress	RO4	My workload is too heavy.
Role stress	RO5	My workload is so heavy that I cannot ensure the quality of my work.
Emotional exhaustion	E1	Work leaves me feeling physically and emotionally depleted.
Emotional exhaustion	E2	I feel drained at the end of each working day.
Emotional exhaustion	E3	I feel tired when I get up in the morning and have to face another day of work.
Emotional exhaustion	E4	Working throughout the day places a heavy strain on me.
Emotional exhaustion	E5	I feel burned out by my work.
Overall social support	EMS1	When I encounter difficulties at work, there is someone who stands by me.
Overall social support	EMS2	When I encounter difficulties at work, someone offers comfort and encouragement.
Overall social support	EMS3	When I encounter difficulties at work, someone listens while I discuss my private feelings.
Overall social support	EMS4	When I encounter difficulties at work, someone shows interest in and concern for my well-being.
Overall social support	IFS1	When I need help at work, someone offers suggestions.
Overall social support	IFS2	When I encounter a work problem, someone provides information that helps me address it.
Overall social support	IFS3	When I encounter difficulties at work, someone helps me identify the cause and offers suggestions.
Work engagement	Y1	I feel full of energy at work.
Work engagement	Y2	At work, I feel strong and vigorous.
Work engagement	Y3	I am enthusiastic about my work.
Work engagement	Y4	My work inspires me.
Work engagement	Y5	When I get up in the morning, I feel like going to work.
Work engagement	Y6	I feel happy when I am working intensely.
Work engagement	Y7	I am proud of the work that I do.
Work engagement	Y8	I am immersed in my work.
Work engagement	Y9	I get carried away when I am working.

Note: All items were rated on a five-point Likert scale (1 = strongly disagree, 5 = strongly agree). The English wording reflects the food-delivery context used in the survey. Higher scores indicate higher levels of the corresponding construct.

## References

[B1-behavsci-16-01392] Chen L. (2020). Labor order under digital control: A study of labor control among food-delivery workers. Sociological Studies.

[B2-behavsci-16-01392] Cobb S. (1976). Social support as a moderator of life stress. Psychosomatic Medicine.

[B3-behavsci-16-01392] Cui N., Hu Y., Xu L. (2014). Role stress in organizations: An integrated framework and future directions. Soft Science.

[B4-behavsci-16-01392] Demerouti E., Bakker A. B., Nachreiner F., Schaufeli W. B. (2001). The job demands-resources model of burnout. Journal of Applied Psychology.

[B5-behavsci-16-01392] Deng H., Guo W., Sheng Y., Zhang W. (2024). A dual-pathway model linking algorithmic management to employee work engagement. Academy of Management Proceedings.

[B6-behavsci-16-01392] Duggan J., Sherman U., Carbery R., McDonnell A. (2020). Algorithmic management and app-work in the gig economy: A research agenda for employment relations and HRM. Human Resource Management Journal.

[B7-behavsci-16-01392] Fassott G., Henseler J., Coelho P. S. (2016). Testing moderating effects in PLS path models with composite variables. Industrial Management & Data Systems.

[B8-behavsci-16-01392] Fornell C., Larcker D. F. (1981). Evaluating structural equation models with unobservable variables and measurement error. Journal of Marketing Research.

[B9-behavsci-16-01392] Gilboa S., Shirom A., Fried Y., Cooper C. (2008). A meta-analysis of work demand stressors and job performance: Examining main and moderating effects. Personnel Psychology.

[B10-behavsci-16-01392] Hair J. F., Hult G. T. M., Ringle C. M., Sarstedt M. (2022). A primer on partial least squares structural equation modeling (PLS-SEM).

[B11-behavsci-16-01392] Hair J. F., Risher J. J., Sarstedt M., Ringle C. M. (2019). When to use and how to report the results of PLS-SEM. European Business Review.

[B12-behavsci-16-01392] Henseler J., Ringle C. M., Sarstedt M. (2015). A new criterion for assessing discriminant validity in variance-based structural equation modeling. Journal of the Academy of Marketing Science.

[B13-behavsci-16-01392] Kock N. (2015). Common method bias in PLS-SEM: A full collinearity assessment approach. International Journal of e-Collaboration.

[B14-behavsci-16-01392] Koeske G. F., Koeske R. D. (1993). A preliminary test of a stress-strain-outcome model for reconceptualizing the burnout phenomenon. Journal of Social Service Research.

[B15-behavsci-16-01392] Lazarus R. S., Folkman S. (1984). Stress, appraisal, and coping.

[B16-behavsci-16-01392] Li C., Shi K. (2003). The influence of distributive and procedural justice on job burnout. Acta Psychologica Sinica.

[B17-behavsci-16-01392] Li C., Zhang Y. (2009). The impact of role stressors on teachers’ physical and mental health. Psychological Development and Education.

[B18-behavsci-16-01392] Li X., Xia Y. (2022). The double-edged sword effect of paradoxical leadership on employee adaptive performance: The roles of work vitality and role stress. Soft Science.

[B19-behavsci-16-01392] Liang B., Wang Y., Huo W., Song M., Shi Y. (2025). Algorithmic control as a double-edged sword: Its relationship with service performance and work well-being. Journal of Business Research.

[B20-behavsci-16-01392] Liang T. P., Ho Y. T., Li Y. W., Turban E. (2011). What drives social commerce: The role of social support and relationship quality. International Journal of Electronic Commerce.

[B21-behavsci-16-01392] Maslach C., Schaufeli W. B., Leiter M. P. (2001). Job burnout. Annual Review of Psychology.

[B22-behavsci-16-01392] Mbare B., Perkiö M., Koivusalo M. (2024). Algorithmic management, well-being and platform work: Understanding the psychosocial risks and experiences of food couriers in Finland. Labour & Industry.

[B23-behavsci-16-01392] Nie T., Qiu T. (2019). Formation of cyberloafing behavior based on the stressor-emotion model. Chinese Journal of Management.

[B24-behavsci-16-01392] Pei J., Liu S., Cui X., Qu J. (2021). Gig workers’ perceived algorithmic control: Conceptualization, measurement, and validation of service performance impact. Nankai Business Review.

[B25-behavsci-16-01392] Pei J., Liu S., Zhang Z., Xie Y. (2024). Good algorithm, bad algorithm? Overwork among gig workers under algorithmic logic. Journal of Industrial Engineering and Engineering Management.

[B26-behavsci-16-01392] Podsakoff P. M., MacKenzie S. B., Lee J. Y., Podsakoff N. P. (2003). Common method biases in behavioral research: A critical review of the literature and recommended remedies. Journal of Applied Psychology.

[B27-behavsci-16-01392] Rizzo J. R., House R. J., Lirtzman S. I. (1970). Role conflict and ambiguity in complex organizations. Administrative Science Quarterly.

[B28-behavsci-16-01392] Sarstedt M., Hair J. F., Cheah J. H., Becker J. M., Ringle C. M. (2019). How to specify, estimate, and validate higher-order constructs in PLS-SEM. Australasian Marketing Journal.

[B29-behavsci-16-01392] Schaufeli W. B., Bakker A. B., Salanova M. (2006). The measurement of work engagement with a short questionnaire: A cross-national study. Educational and Psychological Measurement.

[B30-behavsci-16-01392] Shmueli G., Sarstedt M., Hair J. F., Cheah J. H., Ting H., Vaithilingam S., Ringle C. M. (2019). Predictive model assessment in PLS-SEM: Guidelines for using PLSpredict. European Journal of Marketing.

[B31-behavsci-16-01392] Sun R., Yuan Y., Zhu Q., Chen L., Zhao K. (2024). The double-edged sword effect of perceived algorithmic control on gig workers’ emotional exhaustion: A legitimacy-judgment perspective. Journal of Systems & Management.

[B32-behavsci-16-01392] Wang C., Zhu C. (2018). Role stress and job burnout among customer-contact employees: The moderating effect of social support. Enterprise Economy.

[B33-behavsci-16-01392] Wang Y., Wang Z., Li J. (2024). Does algorithmic control facilitate platform workers’ deviant behavior toward customers? The ego-depletion perspective. Computers in Human Behavior.

[B34-behavsci-16-01392] Weber M., Remus U., Geiger M., Cram W. A. (2026). Between proactive and reactive coping: How food-delivery workers cope with algorithmic management threats. European Journal of Information Systems.

[B35-behavsci-16-01392] Wood A. J., Graham M., Lehdonvirta V., Hjorth I. (2019). Good gig, bad gig: Autonomy and algorithmic control in the global gig economy. Work, Employment and Society.

[B36-behavsci-16-01392] Yao Z., Zhang X. (2020). Time-pressure consistency and turnover intention: The mediating role of emotional exhaustion. Soft Science.

[B37-behavsci-16-01392] Yu S., Liu S., Gong X., Liu C., Cai W. (2025). Online platform algorithmic control and gig workers’ turnover intention in China: The mediating role of relative deprivation. Journal of Management Science and Engineering.

[B38-behavsci-16-01392] Zhan X., Xia J., Wang T., Zhu Z. (2023). The influence mechanism of algorithmic control on platform workers’ emotional exhaustion. Journal of Management Science.

[B39-behavsci-16-01392] Zhang L., Li J., Mao M. (2024). Perceived algorithmic control and gig workers’ work engagement: A dual-path model based on cognition and emotion. Journal of Business Economics.

[B40-behavsci-16-01392] Zhang L., Ling X., Yang C. (2026). Looking at both sides of algorithmic control and employee well-being: A job demands-resources model. Frontiers in Psychology.

[B41-behavsci-16-01392] Zhu D. H., Sun H., Chang Y. P. (2016). Effect of social support on customer satisfaction and citizenship behavior in online brand communities: The moderating role of support source. Journal of Retailing and Consumer Services.

